# Disruption of Cross-Feeding Inhibits Pathogen Growth in the Sputa of Patients with Cystic Fibrosis

**DOI:** 10.1128/mSphere.00343-20

**Published:** 2020-04-29

**Authors:** Jeffrey M. Flynn, Lydia C. Cameron, Talia D. Wiggen, Jordan M. Dunitz, William R. Harcombe, Ryan C. Hunter

**Affiliations:** a Department of Microbiology & Immunology, University of Minnesota, Minneapolis, Minnesota, USA; b Division of Pulmonary, Allergy, Critical Care, & Sleep Medicine, University of Minnesota, Minneapolis, Minnesota, USA; c Department of Ecology, Evolution, and Behavior, University of Minnesota, Saint Paul, Minnesota, USA; University of Kentucky

**Keywords:** *Pseudomonas aeruginosa*, antibiotics, cross-feeding, cystic fibrosis

## Abstract

A critical limitation in the management of chronic polymicrobial infections is the lack of correlation between antibiotic susceptibility testing (AST) and patient responses to therapy. Underlying this disconnect is our inability to accurately recapitulate the *in vivo* environment and complex polymicrobial communities *in vitro*. However, emerging evidence suggests that, if modeled and tested accurately, interspecies relationships can be exploited by conventional antibiotics predicted to be ineffective by standard AST. As an example, under conditions where Pseudomonas aeruginosa relies on cocolonizing organisms for nutrients (i.e., cross-feeding), multidrug-resistant P. aeruginosa may be indirectly targeted by inhibiting the growth of its metabolic partners. While this has been shown *in vitro* using synthetic bacterial communities, the efficacy of a “weakest-link” approach to controlling host-associated polymicrobial infections has not yet been demonstrated. To test whether cross-feeding inhibition can be leveraged in clinically relevant contexts, we collected sputa from cystic fibrosis (CF) subjects and used enrichment culturing to isolate both P. aeruginosa and anaerobic bacteria from each sample. Predictably, both subpopulations showed various antibiotic susceptibilities when grown independently. However, when P. aeruginosa was cultured and treated under cooperative conditions in which it was dependent on anaerobic bacteria for nutrients, the growth of both the pathogen and the anaerobe was constrained despite their intrinsic antibiotic resistance profiles. These data demonstrate that the control of complex polymicrobial infections may be achieved by exploiting obligate or facultative interspecies relationships. Toward this end, *in vitro* susceptibility testing should evolve to more accurately reflect *in vivo* growth environments and microbial interactions found within them.

**IMPORTANCE** Antibiotic efficacy achieved *in vitro* correlates poorly with clinical outcomes after treatment of chronic polymicrobial diseases; if a pathogen demonstrates susceptibility to a given antibiotic in the lab, that compound is often ineffective when administered clinically. Conversely, if a pathogen is resistant *in vitro*, patient treatment with that same compound can elicit a positive response. This discordance suggests that the *in vivo* growth environment impacts pathogen antibiotic susceptibility. Indeed, here we demonstrate that interspecies relationships among microbiotas in the sputa of cystic fibrosis patients can be targeted to indirectly inhibit the growth of Pseudomonas aeruginosa. The therapeutic implication is that control of chronic lung infections may be achieved by exploiting obligate or facultative relationships among airway bacterial community members. This strategy is particularly relevant for pathogens harboring intrinsic multidrug resistance and is broadly applicable to chronic polymicrobial airway, wound, and intra-abdominal infections.

## Abstract (precis)

Antibiotic efficacy achieved *in vitro* correlates poorly with clinical outcomes after treatment of chronic polymicrobial diseases; if a pathogen demonstrates susceptibility to a given antibiotic in the lab, that compound is often ineffective when administered clinically. Conversely, if a pathogen is resistant *in vitro*, patient treatment with that same compound can elicit a positive response. This discordance suggests that the *in vivo* growth environment impacts pathogen antibiotic susceptibility. Indeed, here we demonstrate that interspecies relationships among microbiotas in the sputa of cystic fibrosis patients can be targeted to indirectly inhibit the growth of Pseudomonas aeruginosa. The therapeutic implication is that control of chronic lung infections may be achieved by exploiting obligate or facultative relationships among airway bacterial community members. This strategy is particularly relevant for pathogens harboring intrinsic multidrug resistance and is broadly applicable to chronic polymicrobial airway, wound, and intra-abdominal infections.

## OBSERVATION

Characterization of bacterial infections is commonly achieved by clinical lab culture, in which targeted pathogens are isolated on nutrient-rich media. In turn, antibiotic selections are based on the MICs of antibiotics required to inhibit pathogen growth under near-ideal conditions. Yet, recent data have challenged the utility of antibiotic susceptibility testing (AST) for cystic fibrosis (CF) and other chronic polymicrobial infections by showing no association between the *in vitro* antibiotic susceptibility of a pathogen and the clinical response of its host (1–4). Many patients are unresponsive to antibiotics targeted at canonical pathogens (e.g., Pseudomonas aeruginosa) cultured from clinical samples, while others respond positively to therapies predicted to fail by AST. The inadequacy of predictive diagnostics to inform antibiotic selection remains a critical challenge for medical management. In fact, despite CF treatment guidelines long supporting the use of AST (5, 6), recent data suggest a decline in clinician reliance on *in vitro* susceptibility data to guide treatment decisions (7).

An inherent limitation of AST is the inability to recapitulate the *in vivo* environment *ex vivo*. Within the lung, microbiota encounter a dynamic chemical milieu that varies dramatically from that of laboratory monoculture. For example, oxygen gradients throughout airway mucus affect drug pharmacokinetics and alter bacterial physiology (8), while immune-mediated stress can drive slow-growth phenotypes that augment intrinsic antibiotic resistance (9, 10). Pathogens also thrive as part of a complex polymicrobial community, the interactions of whose members potentiate drug tolerance (11, 12). Accurately modeling the *in vivo* environment and microbial interactions during AST will likely offer opportunities for the development of new therapeutic strategies.

**FIG 1:**
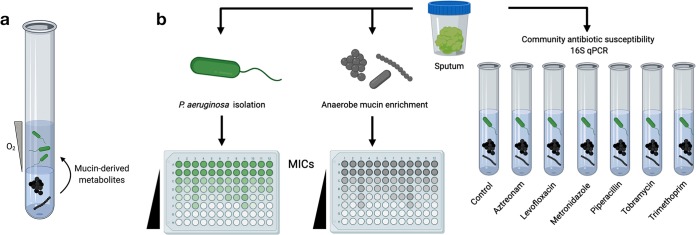
(a) Mucin-based cross-feeding model of CF microbiota. P. aeruginosa (upper portion of solution) is grown in agar without bioavailable nutrients. Anaerobes (lower portion of solution) are grown with mucin as a carbon source, liberating small metabolites for P. aeruginosa growth. In turn, P. aeruginosa creates an oxygen gradient allowing anaerobes to thrive. Under these cooperative conditions, inhibition of anaerobes with antibiotics to which P. aeruginosa is intrinsically resistant constrains overall community growth (13). (b) Antibiotic MICs were determined for both P. aeruginosa and anaerobic consortia derived from patient samples. The effect of community growth on antibiotic susceptibility (i.e., the weakest link) was then determined using our mucin coculture model. 16S rRNA gene sequencing and qPCR were used to quantify and characterize sputum bacterial community growth in response to each antibiotic.

For example, among species that rely on one another for metabolites (termed “cross-feeding”), interdependence renders bacterial communities highly susceptible to failure relative to their constituent species living independently. This was demonstrated using a synthetic bacterial consortium engineered to be mutualistic (cooperative) or competitive, depending on the nutritional environment (13). When the consortium was cooperative, community tolerance (an antibiotic concentration that inhibits overall community growth) was set by the least-tolerant member (“weakest link”). This was also shown using a model CF-relevant consortium of P. aeruginosa and anaerobic bacteria commonly detected by culture and culture-independent sequencing (14, 15). When cocultured *in vitro* on mucin as a carbon source, P. aeruginosa establishes an O_2_ gradient allowing anaerobes to grow, which in turn liberate metabolites that P. aeruginosa cannot obtain from mucin on its own (Fig. 1a). Under these conditions, despite the intrinsic resistance of P. aeruginosa to ampicillin, ampicillin inhibition of the anaerobic subpopulation constrains P. aeruginosa growth. These data suggest that targeting weakest links among interdependent microbiotas may be a viable strategy for inhibiting pathogens *in vivo*; however, whether this approach holds true for patient-derived microbial communities remains to be determined.

## 

### Experimental results.

**FIG 2:**
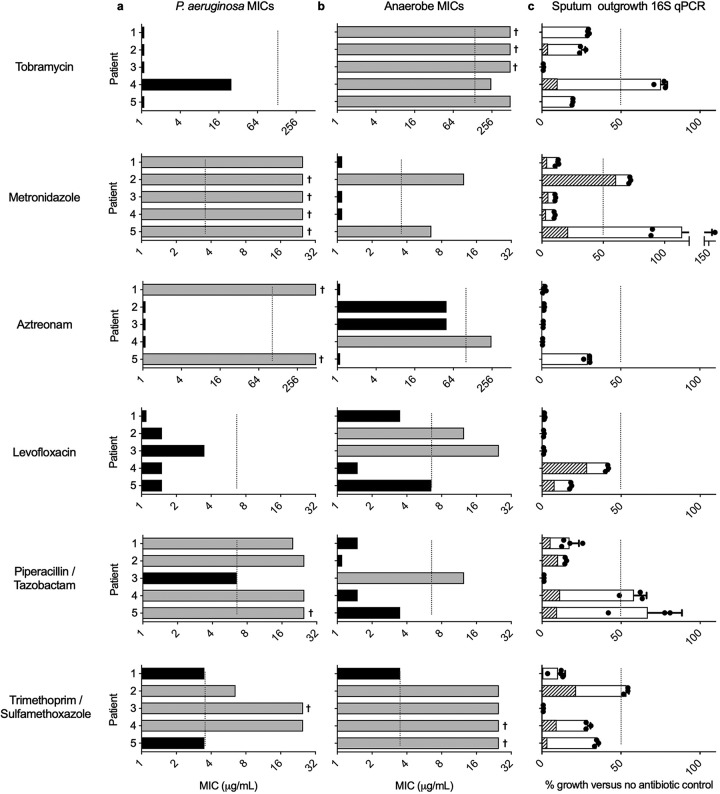
Antibiotic susceptibilities of CF-derived bacterial pathogens are impacted by cooperative community interactions. MICs of six antibiotics were determined for P. aeruginosa (a) and anaerobic bacterial enrichments isolated from sputum (b). Daggers indicate bacterial growth at the highest concentration of antibiotic tested. Bar shading represents the predicted growth (gray) or predicted lack of growth (black) of each subpopulation in the coculture tube assay (c). Growth predictions were determined by comparing individual MICs to the preselected concentration of antibiotic used in the coculture assay (indicated by a dashed line). (c) qPCR was used to quantify the growth of sputum bacterial communities from which P. aeruginosa and anaerobes were derived in the presence of each antibiotic. Growth is expressed as the percent 16S rRNA gene copy number relative to the copy number of an untreated control. Community growth was considered impaired if total 16S rRNA gene copies were less than 50% of the control (dashed line). Data shown are mean qPCR measures from at least three replicate measurements for each coculture tube.

To test the weakest-link approach in a clinically relevant context, we collected sputa from CF patients and isolated P. aeruginosa from each sample (Fig. 1b). The MICs of every antibiotic (tobramycin, aztreonam, levofloxacin, piperacillin-tazobactam [P-T], trimethoprim-sulfamethoxazole [T-S], and metronidazole) were then determined for each isolate under nutrient-rich conditions (akin to the standard AST procedures) (Fig. 2a). Concurrently, we isolated anaerobic mucin-degrading bacteria from each sample (14), and MICs were similarly determined (Fig. 2b). Finally, small aliquots of the remaining sputum homogenate were split between culture tubes containing mucin as the sole carbon source, 1% agarose, and one antibiotic. Using these cultures in which P. aeruginosa and anaerobic mucin degraders were codependent on mucin utilization, we interrogated how cooperative growth affected antibiotic sensitivity relative to that of independent culture (Fig. 2c).

Tobramycin and levofloxacin were broadly effective against P. aeruginosa, while some isolates were resistant to aztreonam, P-T, and T-S (Fig. 2a). Metronidazole was ineffective against aerobic P. aeruginosa, as expected. For anaerobic enrichments (Fig. 2b), nearly all compounds, including those not recognized for anaerobic coverage (aztreonam, levofloxacin), showed various efficacies, the exception being tobramycin, to which all anaerobes were resistant.

We then asked whether antibiotic sensitivity of the overall sputum bacterial community would correspond to the least tolerant among P. aeruginosa and anaerobic subpopulations. To do so, total 16S rRNA gene copy numbers (Fig. 2c) and 16S rRNA gene sequencing (Fig. S1) were used to assess sputum bacterial community growth and composition, respectively, in response to each antibiotic. We first tested two compounds with contrasting efficacies, tobramycin (efficacy against P. aeruginosa) and metronidazole (coverage for anaerobic organisms). Both compounds showed striking effects on cooperative cultures. In general, relative to that of untreated controls, community growth was constrained if either P. aeruginosa or anaerobic subpopulations were susceptible when grown independently. Conversely, when both subpopulations were independently resistant (e.g., metronidazole for patients 2 and 5) or showed intermediate susceptibility (tobramycin for patient 4), community growth was also robust.

10.1128/mSphere.00343-20.2 FIG S1 16S rRNA gene sequencing of sputum bacterial communities in response to antibiotic treatment. Data shown are the bacterial community compositions (relative abundances) of the original sputum sample, an untreated aliquot of that sample grown in a stratified culture system *in vitro*, and six additional aliquots treated with individual antibiotics for 48 h. Bactrim, trimethoprim-sulfamethoxazole; Zosyn, piperacillin-tazobactam. Download FIG S1, JPG file, 1.4 MB. Copyright © 2020 Flynn et al. 2020 Flynn et al. This content is distributed under the terms of the Creative Commons Attribution 4.0 International license.

For aztreonam, levofloxacin, P-T, and T-S, the presence of one antibiotic-susceptible subpopulation was also largely predictive of community failure when the culture was treated with that same compound. For example, two aztreonam-resistant P. aeruginosa isolates were restricted in coculture, likely due to the aztreonam susceptibility of their anaerobic mutualists. However, notable exceptions were also observed, particularly for P-T, which showed varied effects (i.e., cocultures were unaffected despite a susceptible subpopulation). In two instances, community growth was surprisingly inhibited despite the predicted inefficacy of the antibiotic based on individual MICs (T-S for patients 3 and 4). As expected, community growth was always inhibited (i.e., <50%) when both P. aeruginosa and anaerobes were independently susceptible.

### Discussion.

These data demonstrate clear community effects on antibiotic efficacy and underscore the finding that pathogen susceptibility is defined not only by its intrinsic resistance but also on the basis of its growth environment and cocolonizing microbiota. It has been widely documented that antibiotic-sensitive bacteria can elude antibiotic killing *in vivo* as a result of coexisting antibiotic-resistant organisms (e.g., via production of extended-spectrum β-lactamases) (12). Similarly, P. aeruginosa and Staphylococcus aureus have been shown to stimulate vancomycin resistance in Streptococcus anginosus via alteration of its cell wall (16). Such interactions are commonly put forth as explanations for why antibiotics fail clinically despite demonstrated efficacy *in vitro*. Here, we present the converse effect: resistant pathogens can be indirectly suppressed by inhibiting cooperative partners. In this context, identifying weakest links among airway microbiota may improve upon therapeutic effectiveness. Moreover, these off-target effects may explain why some antibiotics that are predicted to fail ultimately have beneficial effects on patients that receive them.

There are plausible explanations for the various effects observed. First, independent MICs were determined in broth culture, whereas cooperative growth was tested in a spatially structured gel. It is known that spatial relationships between species can have profound effects on their physiology (17), and it is possible that bacterial distribution was a determinant of the community susceptibility profiles shown here. In addition, differences in community memberships between subjects or within a heterogeneous sputum sample may explain disparities for a given antibiotic. Specifically, different anaerobic species and other canonical pathogens excrete unique metabolites and degradative enzymes and modify the growth environment in ways in which they can differentially affect antibiotic efficacy. Ongoing work is focused on how community membership, spatial structure, and various antibiotic concentrations affect the community susceptibility phenotypes.

Given the diversity of polymicrobial interactions found at sites of infection, a weakest-link approach is broadly applicable beyond mucin-based cross-feeding and CF airway disease. In theory, any bacterial community in which two or more members exchange metabolites, potentiate virulence, or rely on others for niche modification (e.g., oxygen consumption) could be targeted. As these interactions are more clearly defined and new community relationships are identified, novel therapies are likely to emerge. Such strategies may be critical for emerging pathogens whose innate multidrug resistance poses a significant barrier to direct treatment.

### Sputum collection.

Sputa were collected from adult participants with CF and processed within 30 min of collection. Studies were approved by the UMN Institutional Review Board (protocol number 1511M8052).

### Pseudomonas aeruginosa isolation.

One hundred microliters of sputum was streaked onto *Pseudomonas* isolation agar and incubated at 37°C for 72 h. A single isolate from each sample was screened by PCR to confirm its identity and stored in 30% glycerol at –80°C.

### Anaerobic enrichment.

Sputum was passed into a Coy anaerobic chamber (95% N_2_, 5% CO_2_). Equal volumes of minimal mucin medium (MMM) (13) and phosphate-buffered saline (PBS) were then added, followed by mechanical homogenization using 18-gauge needles. One hundred microliters of each sample was added to 5 ml of MMM containing 15 g/liter purified porcine gastric mucin (13) and incubated at 37°C for 72 h under anoxia to enrich for mucin-degrading bacteria.

### Antibiotic susceptibility testing.

For anaerobic AST, 5 μl of enrichment culture was added to each well of a microtiter plate containing MMM supplemented with gradients of six antibiotics (aztreonam, 0 to 500 μg/ml; levofloxacin, 0 to 25 μg/ml; metronidazole, 0 to 25 μg/ml; piperacillin-tazobactam, 0 to 25 μg/ml; tobramycin, 0 to 500 μg/ml; and trimethoprim-sulfamethoxazole, 0 to 25 μg/ml). Plates were wrapped in Parafilm and incubated anaerobically at 37°C for 72 h. For P. aeruginosa, isolates were grown in lysogeny broth (LB) to an optical density at 600 nm (OD_600_) of 0.5. Five microliters was then added to a similar plate containing LB as the base medium and incubated aerobically at 37°C for 24 h. Cells were resuspended on plates by pipetting, and the isolates were read spectrophotometrically at an OD_600_ using a BioTek Synergy plate reader. MICs were determined based on the antibiotic concentration required to achieve a 90% reduction in culture density relative to that of antibiotic-free controls.

### Coculture.

Under anaerobic conditions, 200 μl of homogenized sputum was placed into one of eight tubes into which a predetermined concentration of each antibiotic was added (aztreonam, 100 μg/ml; levofloxacin, 6.5 μg/ml; metronidazole, 3.5 μg/ml; piperacillin, 6.5 μg/ml, tazobactam, 0.8 μg/ml; tobramycin, 500 μg/ml; trimethoprim, 0.7 μg/ml; sulfamethoxazole, 3.5 μg/ml). Concentrations shown for P-T and T-S refer to piperacillin and sulfamethoxazole, respectively. Concentrations were selected based on results of prior MIC assays demonstrating mixed efficacy (i.e., ∼50% of cells were susceptible) against anaerobic populations isolated from CF sputum. No-antibiotic and cell-free tubes were used as controls. Two hundred microliters of molten 2% low-melting-point agarose in PBS was added and homogenized with a pipette, and each mixture was then added to 0.5-ml Pyrex culture tubes, forming a gel upon cooling. Tubes were incubated aerobically at 37°C for 72 h and frozen at –80°C.

### DNA extraction.

Agar plugs were removed from the culture tubes and transferred to Matrix E lysing tubes (MP Biomedicals), and 2 volumes of lysis buffer was added. Tubes were vortexed on a bead beater twice for 30 s, followed by centrifugation at 9,500 × *g* for 1 min. The supernatant was transferred to a new tube, incubated at 75°C for 10 min, and returned to room temperature. Lysozyme and RNase A were then added to final concentrations of 0.2 mg/ml and 50 μg/ml, respectively, and the tubers were incubated for 1 h at 37°C. One hundred microliters of 10% SDS and proteinase K (2 mg/ml) was added, and the tubes were incubated overnight at 55°C.

DNA was extracted by adding 1 volume of phenol-chloroform-isoamyl alcohol (25:24:1) and vortexing the tube to emulsify the solution. Samples were centrifuged at 21,000 × *g* for 20 min, and the upper aqueous layer was transferred to a new tube. One volume of chloroform-isoamyl alcohol was then added, followed by centrifugation at 21,000 × *g* for 15 min, and the upper layer was retained. Three molar sodium acetate (pH 5.2) was added and mixed gently, followed by the addition of 1 volume of isopropanol and 3 μl GlycoBlue. Samples were centrifuged for 20 min at 21,000 × *g*, and the supernatant was removed. Pellets were washed using 80% ethanol, air dried, and resuspended in 10 mM Tris (pH 8) prior to storage at –80°C.

### qPCR.

Community growth was estimated by quantifying 16S rRNA gene copies from coculture DNA. Reaction mixtures were prepared in triplicate using iTaq Universal SYBR green supermix (Bio-Rad) and standard 16S rRNA gene quantitative PCR (qPCR) primers 338F and 518R (18). Each reaction mixture contained 10 μl 2× SYBR green, 2 μl each of 3 μM primers, and 5 μl H_2_O. DNA from each sample was diluted to 10 ng/μl, and 1 μl was added to each reaction mixture. Amplification was performed using a CFX96 PCR system (Bio-Rad) with the following cycling conditions: 95°C for 5 min, followed by 40 cycles of 95°C for 5 s and 60°C for 30 s. Quantification cycle values were calculated using instrument software (CFX Manager, v. 3.1). Standard curves with a range from 5 × 10^6^ to 5 × 10^2^ 16S rRNA gene copy numbers were prepared using serial dilutions of DNA from Fusobacterium nucleatum ATCC 25586. Community growth was expressed as a percentage of the 16S rRNA gene copy number of untreated controls.

### Data availability.

Genomic DNA was submitted to the UMN Genomics Center (UMGC) for 16S rRNA library preparation as previously described (19). Detailed sequencing and analysis methods can be found in Text S^1^ in the supplemental material. Raw gene sequence data are available as fastq files in the NCBI Sequence Read Archive under BioProject ID PRJNA623678.

10.1128/mSphere.00343-20.1 TEXT S1 Supplemental materials and methods. Download Text S1, DOCX file, 0.01 MB. Copyright © 2020 Flynn et al. 2020 Flynn et al. This content is distributed under the terms of the Creative Commons Attribution 4.0 International license.
