# Supplementary figures and images for: Disruption of Cross-Feeding Inhibits Pathogen Growth in the Sputa of Patients with Cystic Fibrosis
Source: mSphere. 2020 Apr 29;5(2):e00343-20. doi: 10.1128/mSphere.00343-20 (PMC7193046; doi:10.1128/mSphere.00343-20)

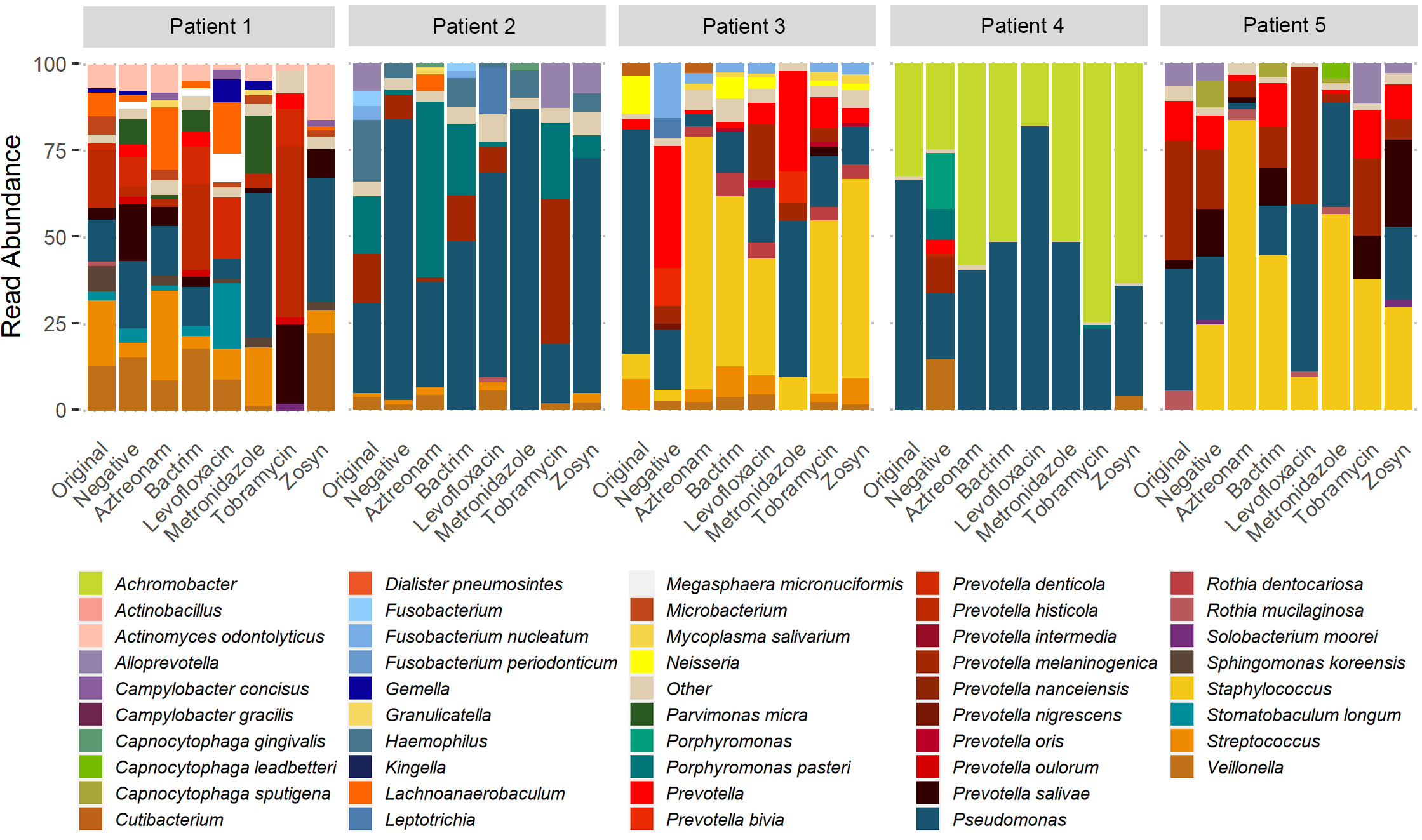

Supplement: FIG S1 [file mSphere.00343-20-sf001.jpg]
