# Supplementary material for: Disruption of Cross-Feeding Inhibits Pathogen Growth in the Sputa of Patients with Cystic Fibrosis
Source: mSphere. 2020 Apr 29;5(2):e00343-20. doi: 10.1128/mSphere.00343-20 (PMC7193046; doi:10.1128/mSphere.00343-20)
Supplement: TEXT S1 [file mSphere.00343-20-s0001.docx]

**Supplemental Materials and Methods**

**Disruption of cross-feeding inhibits pathogen growth in cystic fibrosis sputum**

**Flynn et al., 2020**

**16S rRNA gene sequencing.** Genomic DNA (gDNA) was submitted to the University of Minnesota Genomics Center (UMGC) for 16S rRNA gene library preparation using a two-step PCR protocol as previously described (1). The V4 region was amplified and sequenced on an Illumina MiSeq using TruSeq (v.3) 2x300 paired-end technology. Sequence quality was assessed using DADA2 (2) in R. Cutadapt (3) was used to remove primer and Illumina adapter sequences, with size filtering set to a minimum and maximum of 215bp and 285 bp, respectively. DADA2 functions were used to trim and filter sequences, model and correct Illumina sequence errors, align paired-end sequences, and filter chimeric reads. Forward and reverse sequences were trimmed to 250bp and 200bp, respectively, and a post-trimming minimum length filter of 175bp was applied. All other DADA2 parameters were run using default options. Amplicon sequence variants (ASVs) were assigned taxonomy using RDP classifier (4) and the SILVA SSU database (Release 132, Dec. 2017)(5,6). A phylogenetic tree was approximated using the phangorn R package (7) and sequences were aligned using DECIPHER. The phangorn package was then used to construct a neighbor-joining tree, which was then used to fit a GTR+G+I maximum likelihood tree. ASV count data and taxonomic assignment were performed within the analysis framework of the Phyloseq R package (v.1.26)(8). Custom R code used in sequence analysis is available on Github (https://github.umn.edu/valen278/Cameron_2020). Raw 16S rRNA gene sequencing data are available as fastq files in the NCBI sequence read archive under Bioproject ID PRJNA623678.

**Supplemental References**

1. Gohl DM, Vangay P, Garbe J, MacLean A, Hauge A, Becker A, Gould TJ, Clayton JB, Johnson TJ, Hunter R, Knights D, Beckman KB. 2016. Systematic improvement of amplicon marker gene methods for increased accuracy in microbiome studies. Nat Biotechnol 34:942-949.
2. Callahan BJ, McMurdie PJ, Rosen MJ, Han AW, Johnson AA, Holmes SP. 2016. DADA2: High-resolution sample inference from Illumina amplicon data. Nat Methods 13:581-583.
3. Martin M. 2011. Cutadapt removes adapter sequences from high-throughput sequencing reads. EMBnet J 1:10-12.
4. Wang Q, Garrity GM, Tiedje JM, Cole JR. 2007. Naive Bayesian classifier for rapid assignment of rRNA sequences into the new bacterial taxonomy. Appl Environ Microbiol 73:5261-5267.
5. Yilmaz P, Wegener Parfrey L, Yarza P, Gerken J, Pruesse E, Quast C, Schweer T, Peplies J, Ludwig W, Glockner FO. 2014. The SILVA and “all-species living tree project (LTP)” taxonomic frameworks. Nucl Acids Res 42:D643-D648.
6. Quast C, Pruesse E, Yilmaz P, Gerken J, Schweer T, Yarza P, Peplies J, Glockner FO. 2013. The SILVA ribosomal RNA gene database project: improved data processing and web-based tools. Nucl Acids Res 41:D590-D596.
7. Schliep KP. 2011. phangorn: phylogenetic analysis in R. Bioinformatics 27:592-593.
8. McMurdie PJ, Holmes S. 2013. phyloseq: an R package for reproducible interactive analysis and graphics of microbiome census data. PLoS One 8:e61217.
